# Supplementary material for: End Sequence Analysis Toolkit (ESAT) expands the extractable information from single-cell RNA-seq data
Source: Genome Res. 2016 Oct;26(10):1397–410. doi: 10.1101/gr.207902.116 (PMC5052061; doi:10.1101/gr.207902.116)
Supplement: Supplemental Material [file supp_gr.207902.116_Supp_Materials.tar.gz › ESAT-esat_v0.1/doc/html/hierarchy.html]

ESAT: Class Hierarchy


ESAT


- Main Page
- Related Pages
- Packages
- Classes
- Files

- Class List
- Class Index
- Class Hierarchy
- Class Members


All Classes Namespaces Files Functions Variables Enumerator Pages

Class Hierarchy

Go to the graphical class hierarchy

This inheritance list is sorted roughly, but not completely, alphabetically:

[detail level 123456]

|  |  |
| --- | --- |
| ►C**AbstractAnnotationCollection** |  |
| Cumms.core.annotation.AnnotationList< T extends Annotation > |  |
| Cumms.core.model.AlignmentModel |  |
| Cumms.core.model.JCSAlignmentModel |  |
| ►Cbroad.core.sequence.AbstractFastaParser |  |
| Cbroad.core.sequence.FastaParser |  |
| Cbroad.core.sequence.FastaQualityParser |  |
| ►C**AbstractWindowScore** |  |
| Cbroad.pda.seq.rap.SkellamScore |  |
| Cbroad.pda.geneexpression.agilent.AgilentArrayStats |  |
| Cbroad.pda.geneexpression.agilent.AgilentProbe |  |
| Cbroad.pda.geneexpression.agilent.AgilentToGCT |  |
| Cbroad.pda.geneexpression.agilent.AgilentUtils |  |
| Cjaligner.Alignment |  |
| Cumms.core.model.AlignmentModel.AlignmentCount |  |
| Cumms.core.model.JCSAlignmentModel.AlignmentCount |  |
| ►Cbroad.pda.seq.segmentation.AlignmentDataModel |  |
| Cbroad.pda.seq.segmentation.GenericAlignmentDataModel |  |
| Cbroad.pda.seq.segmentation.AlignmentDataModelStats |  |
| Cbroad.core.multiplealignment.AlignmentExtractor |  |
| Cbroad.pda.seq.alignment.AlignmentUtils |  |
| Cbroad.core.motif.meme.AMEResultReader |  |
| Cumms.core.annotation.AnnotationCollection< T extends Annotation > |  |
| Cbroad.pda.countreads.AnnotationCounts |  |
| Cbroad.core.annotation.AnnotationFactoryFactory |  |
| Cumms.core.annotation.AnnotationFileReader |  |
| ►Cbroad.core.annotation.AnnotationHandler |  |
| Cbroad.pda.datastructures.LocationAwareMatrix |  |
| Cbroad.core.annotation.AnnotationReader< T extends GenomicAnnotation > |  |
| Cbroad.core.annotation.AnnotationReaderFactory |  |
| Cumms.core.utils.AnnotationUtils |  |
| Cbroad.core.annotation.AnnotationUtils |  |
| Cbroad.core.math.ANOVA |  |
| Cbroad.core.siphy.tools.conservation.AverageBranchLength |  |
| Cbroad.pda.differentialExpression.BatchNorm |  |
| Cbroad.core.siphy.tools.conservation.BatchSubmitEstimateExon |  |
| ►Cbroad.pda.annotation.BEDFileParser |  |
| Cbroad.pda.annotation.GTFFileParser |  |
| Cumms.core.scripture.BuildScriptureCoordinateSpace |  |
| Cjaligner.Cell |  |
| Cbroad.pda.feature.genome.Chromosome |  |
| Cbroad.pda.differentialExpression.CisVsTrans |  |
| Cbroad.pda.geneexpression.agilent.ClassifyAgilentLincProbes |  |
| ►C**Cloneable** |  |
| ►Cbroad.core.annotation.GenomicAnnotation |  |
| ►Cbroad.core.annotation.BasicGenomicAnnotation |  |
| Cbroad.core.annotation.BED |  |
| Cbroad.core.annotation.GFF |  |
| Cbroad.core.annotation.RepeatMaskerAnnotation |  |
| ►Cbroad.core.annotation.ShortBED |  |
| Cbroad.core.annotation.BEDGraph |  |
| Cbroad.pda.datastructures.Alignments |  |
| CJama.Matrix |  |
| Cbroad.pda.geneexpression.clustering.Cluster |  |
| ►Cbroad.pda.geneexpression.clustering.ClusterDistanceFunction |  |
| Cbroad.pda.geneexpression.clustering.AbsolutePearsonFunction |  |
| Cbroad.pda.geneexpression.clustering.EuclideanDistance |  |
| Cbroad.pda.geneexpression.clustering.PearsonDistance |  |
| Cbroad.core.util.CLUtil |  |
| Cbroad.core.util.CollapseByIntersection |  |
| Cumms.esat.CollapsedGeneLengths |  |
| Cbroad.pda.geneexpression.agilent.CollapseProbesByMinPValue |  |
| Cbroad.pda.differentialExpression.CollapseProjections |  |
| ►C**CollectRnaSeqMetrics** |  |
| Cbroad.pda.seq.rap.rna.CollectRnaSeqMetrics |  |
| Cbroad.core.math.CombinationGenerator |  |
| Cbroad.core.parser.CommandLineParser |  |
| Cjaligner.util.Commons |  |
| ►C**Comparable** |  |
| Cbroad.core.primer3.HairpinKmer |  |
| Cbroad.pda.seq.graph.Path |  |
| Cumms.core.alignment.FeatureSequenceAlignment.UnorderedGenePair |  |
| ►Cumms.core.annotation.Annotation |  |
| ►Cbroad.core.annotation.LightweightGenomicAnnotation |  |
| ►Cbroad.core.annotation.BasicLightweightAnnotation |  |
| Cbroad.core.annotation.BasicGenomicAnnotation |  |
| Cbroad.core.annotation.GenomicAnnotation |  |
| ►Cumms.core.alignment.Alignment |  |
| ►Cumms.core.alignment.AbstractPairedEndAlignment |  |
| Cumms.core.alignment.FragmentAlignment |  |
| Cumms.core.alignment.PairedReadAlignment |  |
| Cumms.core.alignment.SingleEndAlignment |  |
| ►Cumms.core.annotation.AbstractAnnotation |  |
| ►Cumms.core.annotation.BasicAnnotation |  |
| Cumms.core.alignment.AbstractPairedEndAlignment |  |
| Cumms.core.alignment.SingleEndAlignment |  |
| ►Cumms.core.annotation.Gene |  |
| Cumms.core.feature.GeneWindow |  |
| Cumms.core.annotation.PSLRecord |  |
| Cumms.core.feature.GenomeWindow |  |
| ►Cumms.core.feature.Window |  |
| Cumms.core.feature.GeneWindow |  |
| Cumms.core.feature.GenomeWindow |  |
| Cumms.core.annotation.CompoundInterval |  |
| Cumms.core.annotation.SingleInterval |  |
| ►Cumms.core.model.score.CountScore |  |
| Cumms.core.model.score.BasicScanStatisticScore |  |
| Cumms.core.model.score.BinomialEnrichmentScore |  |
| Cumms.core.model.score.MultiScore |  |
| Cumms.core.model.score.NewRatioScore |  |
| Cumms.core.model.score.PoissonEnrichmentScore |  |
| Cumms.core.model.score.RatioScore |  |
| Cumms.core.model.score.ScanStatisticScore |  |
| Cumms.core.model.score.WindowAllScore |  |
| Cumms.core.model.score.EndInWindowCountScore |  |
| Cumms.core.model.score.StartInWindowCountScore |  |
| Cbroad.core.motif.misc.ComputeConservedMotifs |  |
| Cbroad.pda.geneexpression.clustering.ComputeCorrelationOfReplicates |  |
| Cbroad.pda.seq.chip.ComputeEnrichments |  |
| Cbroad.core.math.ComputeFDR |  |
| Cbroad.pda.geneexpression.ComputeHypergeomtricEnrichment |  |
| Cbroad.core.siphy.tools.conservation.ComputeKmersForRegion |  |
| Cbroad.core.siphy.tools.conservation.ComputeMaxPercentileOfElements |  |
| Cbroad.core.primer3.ComputeMIRScore |  |
| Cbroad.core.primer3.ComputeOriginalScore |  |
| Cbroad.core.siphy.tools.conservation.ComputePercentageOfKmers |  |
| Cumms.core.scripture.statistics.ConnectDisconnectedTranscripts |  |
| Cbroad.core.siphy.ConservationUtils |  |
| Cbroad.core.siphy.ConservedPWMScanner |  |
| Cbroad.pda.seq.segmentation.ContinuousDataAlignmentModel |  |
| Cbroad.pda.differentialExpression.ConvertToDiscreteMatrix |  |
| ►Cumms.core.coordinatesystem.CoordinateSpace |  |
| Cumms.core.coordinatesystem.GenomicSpace |  |
| Cumms.core.coordinatesystem.MaskedGenomicSpace |  |
| Cumms.core.coordinatesystem.TranscriptInGenomicSpace |  |
| Cumms.core.coordinatesystem.TranscriptomeSpace |  |
| Cbroad.pda.geneexpression.agilent.CorrelationAgilentNanostring |  |
| Cbroad.pda.geneexpression.agilent.CorrelationBetweenReplicates |  |
| Cbroad.pda.geneexpression.clustering.CorrelationMatrix |  |
| Cbroad.pda.geneexpression.CountNums |  |
| Cbroad.core.primer3.CrossJunctionPCRDesigner |  |
| Cbroad.projection.gsa.DefineGeneSignatures |  |
| Cbroad.pda.seq.segmentation.DefineSourceAndSinkFromPairedEnds |  |
| Cbroad.pda.differentialExpression.DifferentialExpression |  |
| Cbroad.pda.differentialExpression.DifferentialScoring |  |
| Cjaligner.Directions |  |
| Cbroad.pda.feature.genome.DirectoryInstalledGenomeAssembly |  |
| Cbroad.core.math.Distribution |  |
| Cbroad.pda.seq.graph.ChromosomeWithBubblesJGraphT.EdgeSourceType |  |
| Cbroad.core.math.EmpiricalDistribution |  |
| Cbroad.pda.enrichment.EnrichmentMetric |  |
| Cbroad.pda.enrichment.EnrichmentUtils |  |
| Cumms.esat.ESAT |  |
| Cumms.esat.ESATDistanceCalculator |  |
| Cbroad.core.siphy.tools.conservation.EstimateOmegaPerExon |  |
| Cbroad.core.siphy.EvolutionaryModel |  |
| Cbroad.core.siphy.EvolutionaryModelParameters |  |
| ►C**Exception** |  |
| Cbroad.core.datastructures.IllegalTreeOperationException |  |
| Cbroad.core.hmm.BadModelException |  |
| Cbroad.core.motif.SearchException |  |
| Cbroad.core.primer3.Primer3Exception |  |
| Cbroad.core.siphy.UnableToFitException |  |
| Cjaligner.matrix.MatrixLoaderException |  |
| Cjaligner.util.SequenceParserException |  |
| Cumms.core.alignment.ChromosomeInconsistencyException |  |
| Cumms.core.exception.IncompleteMethodImplementationException |  |
| Cbroad.pda.geneexpression.ExpressionExperimentInfo |  |
| Cbroad.projection.gsa.ExtractGenes |  |
| Cbroad.core.sequence.Extractor |  |
| Cbroad.pda.geneexpression.agilent.ExtractSamplesAndLabel |  |
| Cbroad.pda.rnai.ExtractSequence |  |
| ►C**Factory** |  |
| Cbroad.core.annotation.AnnotationFactory< T extends LightweightGenomicAnnotation > |  |
| Cumms.core.general.TabbedReader.Factory< T > |  |
| Cbroad.pda.seq.clip.FastaExtendPeaks |  |
| ►Cbroad.core.sequence.FastaHandler |  |
| Cbroad.core.sequence.FastaQualityHandler |  |
| Cbroad.core.sequence.FastaQualityBinaryIO |  |
| Cbroad.core.sequence.FastaQualityIndex |  |
| Cbroad.core.sequence.FastaSequenceIO |  |
| Cbroad.pda.countreads.FastqLibraryStats |  |
| Cbroad.pda.seq.fastq.FastqSequence |  |
| Cbroad.pda.seq.fastq.FastqSplitterByBarcode |  |
| Cbroad.core.math.FDRDistribution |  |
| ►Cbroad.core.annotation.Feature |  |
| Cbroad.core.annotation.GenomicAnnotation |  |
| Cumms.core.alignment.FeatureSequenceAlignment |  |
| Cbroad.pda.geneexpression.FilterGCT |  |
| Cbroad.pda.geneexpression.FilterGCTFile |  |
| Cbroad.pda.geneexpression.agilent.FilterMatrixByMinCutoff |  |
| Cbroad.pda.seq.chip.FindDiffrentialEnrichedRegions |  |
| Cbroad.pda.rap.misc.FindEnrichedDepletedRegions |  |
| Cbroad.pda.geneexpression.agilent.FindProbesForGeneSet |  |
| Cbroad.core.math.FisherCombinedProbabilityTest |  |
| Cbroad.core.siphy.Fit |  |
| Cbroad.pda.differentialExpression.FixedControlDifferentialExpression |  |
| Cbroad.pda.differentialExpression.FoldSignificance |  |
| ►Cjaligner.formats.Format |  |
| Cjaligner.formats.CLUSTAL |  |
| Cjaligner.formats.FASTA |  |
| Cjaligner.formats.Pair |  |
| Cjaligner.formats.FormatFactory |  |
| Cbroad.pda.gene.functionalAnnotation |  |
| Cbroad.pda.differentialExpression.FWERDistribution |  |
| Cbroad.pda.seq.segmentation.GeneCounts |  |
| Cbroad.pda.geneexpression.geneExpUtils |  |
| Cbroad.core.datastructures.GenericRootedTree< T > |  |
| Cbroad.projection.gsa.GeneSetEnrichment |  |
| Cbroad.core.overlaputils.GeneSetIntersect |  |
| Cbroad.pda.gene.GeneTools |  |
| Cumms.core.coordinatesystem.TranscriptomeSpace.GeneTree |  |
| Cbroad.pda.seq.segmentation.GenomeWithGaps |  |
| Cbroad.pda.seq.segmentation.GenomeWithGaps2 |  |
| Cbroad.core.annotation.GenomicAnnotationFilter< T extends LightweightGenomicAnnotation > |  |
| Cbroad.pda.seq.segmentation.GlobalSpliceFilter |  |
| Cbroad.pda.geneexpression.GlobalStats |  |
| Cbroad.core.util.GMTParser |  |
| Cbroad.pda.differentialExpression.GrabProbes |  |
| Cbroad.pda.geneexpression.GrabSpecificColumns |  |
| Cbroad.pda.differentialExpression.GSAProjection |  |
| Cbroad.projection.gsa.GSAScoring |  |
| Cbroad.projection.gsa.GsaUtils |  |
| Cbroad.pda.geneexpression.clustering.HierarchicalClustering |  |
| Cbroad.core.datastructures.Interval |  |
| Cbroad.core.datastructures.IntervalTree< V > |  |
| Cbroad.core.primer3.isPCRLike |  |
| ►C**Iterable** |  |
| Cumms.core.annotation.AnnotationList< T extends Annotation > |  |
| Cbroad.core.datastructures.JCSCache |  |
| Cbroad.pda.geneexpression.clustering.Kmeans |  |
| Cbroad.core.math.KSTest |  |
| Cbroad.pda.annotation.Locus |  |
| Cbroad.core.math.Loess |  |
| Cbroad.pda.geneexpression.agilent.MakeCLSFiles |  |
| Cbroad.pda.geneexpression.agilent.MakeCLSFromDescription |  |
| Cbroad.pda.differentialExpression.MakePositionalGeneSets |  |
| Cbroad.core.math.MannWhitney |  |
| Cbroad.core.math.MannWhitneyPermutations |  |
| Cbroad.core.hmm.MarkovModel< T > |  |
| Cbroad.core.hmm.MarkovState< T > |  |
| Cjaligner.Markups |  |
| CJama.util.Maths |  |
| Cbroad.core.math.MathUtil |  |
| Cjaligner.matrix.MatrixGenerator |  |
| ►Cbroad.core.datastructures.MatrixWithHeaders |  |
| Cbroad.pda.datastructures.LocationAwareMatrix |  |
| Cbroad.core.datastructures.MatrixWithHeaderUtils |  |
| Cbroad.core.annotation.MaximumContiguousSubsequence |  |
| Cbroad.core.motif.meme.MemeMotif |  |
| Cbroad.core.motif.meme.MemeResultReader |  |
| Cbroad.pda.differentialExpression.MergeGSEAOutputIntoMatrix |  |
| ►Cbroad.core.multiplealignment.MultipleAlignment |  |
| Cbroad.core.multiplealignment.MAFAlignment |  |
| Cbroad.core.multiplealignment.MultipleAlignmentFactory |  |
| ►Cbroad.core.multiplealignment.MultipleAlignmentIO |  |
| Cbroad.core.multiplealignment.FastaMultipleAlignmentIO |  |
| Cbroad.core.multiplealignment.MAFIO |  |
| Cbroad.core.multiplealignment.PhylipInterleavedMultipleAlignmentIO |  |
| Cbroad.core.multiplealignment.PhylipSequencialMultipleAlignmentIO |  |
| Cbroad.core.multiplealignment.MultipleAlignmentIOFactory |  |
| ►Cbroad.pda.seq.protection.MultiSampleScanPeakCaller |  |
| Cbroad.pda.seq.protection.BatchedMultiSampleScanPeakCaller |  |
| Cjaligner.NeedlemanWunsch |  |
| Cjaligner.NeedlemanWunschGotoh |  |
| Cbroad.pda.gene.NeighborAnalysis |  |
| Cbroad.pda.gene.NeighborsNullModel |  |
| Cbroad.pda.geneexpression.PaGEJava |  |
| Cbroad.core.datastructures.Pair< T1 > |  |
| Cumms.core.alignment.PairedEndAlignmentFactory |  |
| Cumms.core.readers.PairedEndReader |  |
| Cumms.core.writers.PairedEndWriter |  |
| Cbroad.core.util.ParseGCTFile |  |
| Cbroad.pda.seq.chip.PeakFilter |  |
| Cbroad.core.math.PermutationDistribution |  |
| Cbroad.pda.differentialExpression.Permutations |  |
| Cbroad.core.siphy.PhastconsReader |  |
| Cbroad.pda.seq.clip.Pileup |  |
| Cbroad.core.motif.PositionWeightColumn |  |
| Cbroad.core.motif.PositionWeightMatrixIO |  |
| Cbroad.core.siphy.PositionWeightMatrixModel |  |
| Cbroad.pda.seq.segmentation.PrecomputeDataAlignmentStats |  |
| Cumms.core.general.Predicates |  |
| Cbroad.pda.geneexpression.PreprocessDataSet |  |
| Cbroad.core.primer3.Primer3PickingSummary |  |
| Cbroad.core.primer3.Primer3PrimerPickingResult |  |
| Cbroad.core.primer3.PrimerUtils |  |
| Cbroad.pda.geneexpression.agilent.analysis.ProcessDataset |  |
| Cbroad.pda.tools.PSLXToSAM |  |
| Cbroad.core.motif.PWMUtils |  |
| Cbroad.pda.geneexpression.agilent.QCAgilentResults |  |
| Cbroad.pda.geneexpression.clustering.RankOrder |  |
| Cbroad.pda.seq.rap.RAPPeakCaller |  |
| Cbroad.pda.seq.segmentation.ReadCountNormalizer |  |
| Cbroad.pda.seq.segmentation.ReadFilter< T > |  |
| Cbroad.pda.seq.protection.ReadSimulator |  |
| Cbroad.pda.seq.protection.ReadSimulator2 |  |
| Cbroad.pda.seq.segmentation.utils.ReconstructionComparer |  |
| Cbroad.core.primer3.RestrictionEnzyme |  |
| Cbroad.pda.rnai.RNAiGeneAnnotation |  |
| Cbroad.pda.differentialExpression.RunAllGSEABatch |  |
| Cbroad.pda.differentialExpression.RunDifferentialExpression |  |
| Cbroad.pda.differentialExpression.RunGSEA |  |
| ►C**RuntimeException** |  |
| Cbroad.core.error.ParseException |  |
| Cumms.core.coordinatesystem.AnnotationOutOfBoundsException |  |
| Cumms.core.coordinatesystem.PermutationNotFoundException |  |
| Cumms.core.exception.RuntimeIOException |  |
| Cbroad.pda.differentialExpression.RunTimeSeries |  |
| Cbroad.pda.samtools.SAMFragmentWriter |  |
| ►Cbroad.pda.seq.protection.SampleData |  |
| Cbroad.pda.seq.protection.GenomeSpaceSampleData |  |
| ►Cbroad.pda.seq.clip.SampleData |  |
| Cbroad.pda.seq.clip.GenomeSpaceSampleData |  |
| Cbroad.core.siphy.ScaledWindowParserListener |  |
| Cbroad.core.math.ScanStatistics |  |
| Cbroad.pda.geneexpression.ScoreDiffGenes |  |
| Cbroad.core.motif.misc.ScoreMotifInstances |  |
| ►Cbroad.core.sequence.Sequence |  |
| Cbroad.core.sequence.SequenceRegion |  |
| ►Cbroad.core.motif.SequenceMotif |  |
| Cbroad.core.motif.SequenceMotifCluster |  |
| Cjaligner.util.SequenceParser |  |
| Cbroad.core.sequence.SequenceRegionWindow |  |
| Cbroad.core.sequence.SequenceUtils |  |
| ►C**Serializable** |  |
| CJama.CholeskyDecomposition |  |
| CJama.EigenvalueDecomposition |  |
| CJama.LUDecomposition |  |
| CJama.Matrix |  |
| CJama.QRDecomposition |  |
| CJama.SingularValueDecomposition |  |
| Cumms.core.alignment.AbstractPairedEndAlignment |  |
| Cumms.core.alignment.SingleEndAlignment |  |
| Cumms.core.alignment.SingleEndAlignment.WrapSamRecord |  |
| Cumms.core.annotation.BasicAnnotation |  |
| Cumms.core.annotation.CompoundInterval |  |
| Cumms.core.annotation.SingleInterval |  |
| Cumms.core.alignment.SmatchLike |  |
| Cumms.core.alignment.SmithWatermanAlignment |  |
| Cjaligner.SmithWatermanGotoh |  |
| Cbroad.pda.seq.fastq.SplitAndTrimIndex |  |
| Cbroad.pda.differentialExpression.SplitByName |  |
| Cbroad.pda.seq.fastq.SplitFastqByIndex |  |
| Cbroad.core.siphy.tools.conservation.SplitFile |  |
| Cbroad.core.siphy.StationaryDistributionIO |  |
| Cbroad.core.math.Statistics |  |
| Cbroad.pda.seq.alignment.StoredAlignmentStats |  |
| Cumms.core.annotation.Annotation.Strand |  |
| Cbroad.core.parser.StringParser |  |
| Cumms.core.general.TabbedReader |  |
| Cjaligner.TracebackNode |  |
| Cbroad.core.sequence.TranscribedRegions |  |
| Cbroad.core.sequence.TranscribedSequenceComposition |  |
| Cbroad.pda.gene.transcriptsNullModel |  |
| Cbroad.core.siphy.TreeScaler |  |
| Cbroad.core.siphy.TreeScalerIO |  |
| Cbroad.pda.seq.protection.TwoSampleScanSkellamPeakCaller |  |
| ►Cbroad.core.annotation.TwoSubjectAnnotation |  |
| Cbroad.core.annotation.BasicTwoSubjectAnnotation |  |
| Cbroad.core.math.UnfairDie |  |
| Cbroad.core.math.Wilcox2Sample |  |
| Cumms.core.model.score.WindowProcessor< S extends WindowScore > |  |
| ►Cumms.core.model.score.WindowScore |  |
| ►Cumms.core.model.score.WindowScore.AbstractWindowScore |  |
| ►Cumms.core.model.score.AbstractListScore |  |
| Cumms.core.model.score.CoverageScore |  |
| Cumms.core.model.score.LengthScore |  |
| Cumms.core.model.score.SumScore |  |
| Cumms.core.model.score.CountScore |  |
| Cumms.core.model.score.EndInWindowCountScore |  |
| Cumms.core.model.score.StartInWindowCountScore |  |
| Cbroad.pda.seq.protection.WindowScores |  |
| ►C**AlignmentQueryReader** |  |
| Cbroad.pda.samtools.SAMPairedEndFileReader |  |
| ►C**Annotation** |  |
| Cbroad.core.sequence.SequenceRegion |  |
| ►C**AnnotationCollection** |  |
| Cumms.core.annotation.AbstractAnnotationCollection< T extends Annotation > |  |
| ►C**AnnotationReader** |  |
| Cbroad.core.annotation.BasicAnnotationReader |  |
| Cbroad.core.annotation.BasicGenomicAnnotationReader |  |
| Cbroad.core.annotation.BEDGraphReader |  |
| Cbroad.core.annotation.BEDReader |  |
| Cbroad.core.annotation.GFFReader |  |
| Cbroad.core.annotation.RepeatMaskerAnnotationReader |  |
| Cbroad.core.annotation.ShortBEDReader |  |
| Cbroad.pda.datastructures.AlignmentsReader |  |
| ►C**ArrayList** |  |
| Cbroad.core.motif.PositionWeightMatrix |  |
| ►C**BasicAnnotation** |  |
| Cbroad.core.annotation.BasicLightweightAnnotation |  |
| ►C**CloseableIterator** |  |
| Cbroad.core.datastructures.JCSCache.JCSNodeIterator |  |
| Cbroad.pda.samtools.PairedEndReadIterator |  |
| Cbroad.pda.samtools.WrappedIterator |  |
| Cbroad.pda.seq.segmentation.WrappedIGVIterator |  |
| Cumms.core.coordinatesystem.ShuffledIterator< T extends Annotation > |  |
| Cumms.core.general.CloseableFilterIterator< T > |  |
| Cumms.core.model.AlignmentModel.FilteredIterator |  |
| Cumms.core.model.AlignmentModel.NodeIterator |  |
| Cumms.core.model.AlignmentModel.WrapAlignmentCountIterator |  |
| Cumms.core.model.JCSAlignmentModel.FilteredIterator |  |
| Cumms.core.model.JCSAlignmentModel.NodeIterator |  |
| Cumms.core.model.JCSAlignmentModel.WrapAlignmentCountIterator |  |
| Cumms.core.model.score.WindowScoreIterator< T extends WindowScore > |  |
| ►C**Clusterable** |  |
| Cbroad.core.motif.PositionWeightMatrix |  |
| ►C**CommandLineProgram** |  |
| Cbroad.pda.samtools.FilterFastqReads |  |
| Cbroad.pda.samtools.FilterPairedReadsByMAQ |  |
| Cbroad.pda.samtools.PairedEndTDF |  |
| Cbroad.pda.samtools.TrimFastqReads |  |
| Cbroad.pda.seq.rap.CalculateCoverageStats |  |
| Cbroad.pda.seq.rap.CountProbes |  |
| Cbroad.pda.seq.rap.DistanceToBED |  |
| ►Cbroad.pda.seq.rap.GenomeCommandLineProgram |  |
| Cbroad.pda.seq.rap.BuildRatioNullDistribution |  |
| Cbroad.pda.seq.rap.CollectAnnotationEnrichments |  |
| Cbroad.pda.seq.rap.GCContent |  |
| ►Cbroad.pda.seq.rap.GenomeScoringProgram |  |
| Cbroad.pda.seq.rap.CountReads |  |
| Cbroad.pda.seq.rap.PlotAggregateRegions |  |
| Cbroad.pda.seq.rap.SlideAndCalculate |  |
| Cbroad.pda.seq.rap.SlideAndCount |  |
| Cbroad.pda.seq.rap.RatioPermutationPeakCaller |  |
| ►Cbroad.pda.seq.rap.rna.TranscriptomeCommandLineProgram |  |
| ►Cbroad.pda.seq.rap.rna.TranscriptomeScoringProgram |  |
| Cbroad.pda.seq.rap.rna.CountReads |  |
| Cbroad.pda.seq.rap.ShuffleBAM |  |
| Cbroad.pda.seq.rap.ShuffleBED |  |
| Cbroad.pda.seq.rap.SkellamKLPeakCaller |  |
| Cbroad.pda.seq.rap.UnmarkDuplicates |  |
| ►C**Comparator** |  |
| Cbroad.core.util.ChromosomeStringComparator |  |
| Cjaligner.matrix.MatricesCompartor |  |
| ►C**DefaultDirectedGraph** |  |
| Cbroad.pda.seq.graph.ChromosomeWithBubblesJGraphT |  |
| ►C**DefaultDirectedWeightedGraph** |  |
| Cumms.core.scripture.OrientedChromosomeTranscriptGraph |  |
| ►C**Gene** |  |
| Cbroad.pda.gene.GeneWithIsoforms |  |
| Cbroad.pda.seq.segmentation.GeneScore |  |
| ►C**GraphPath** |  |
| Cbroad.pda.seq.graph.Path |  |
| ►C**Iterator** |  |
| Cbroad.core.datastructures.IntervalTree< V >.FwdIterator |  |
| Cbroad.core.datastructures.IntervalTree< V >.OverlapIterator |  |
| Cbroad.core.datastructures.IntervalTree< V >.RevIterator |  |
| Cbroad.core.datastructures.ReversibleIterator< E > |  |
| Cbroad.core.sequence.WindowSlider |  |
| Cbroad.pda.seq.fastq.FastqParser |  |
| ►C**MarkovModel** |  |
| Cbroad.core.siphy.PiHMM |  |
| ►C**Pair** |  |
| Cumms.core.alignment.AlignmentPair |  |
| ►C**Predicate** |  |
| Cnextgen.core.readFilters.GenomicSpanFilter |  |
| Cnextgen.core.readFilters.PairedAndProperFilter |  |
| Cnextgen.core.readFilters.ReadsToReconstructFilter |  |
| Cumms.core.annotation.filter.FullyContainedFilter |  |
| Cumms.core.annotation.filter.OverlapFilter |  |
| Cumms.core.readFilters.MappingQualityFilter |  |
| Cumms.core.readFilters.PairedEndFilter |  |
| Cumms.core.readFilters.PCRDuplicateFilter |  |
| Cumms.core.readFilters.ReadEndsInFilter |  |
| Cumms.core.readFilters.ReadStartsInFilter |  |
| Cumms.core.readFilters.SameOrientationFilter |  |
| Cumms.core.readFilters.SplicedReadFilter |  |
| Cumms.core.readFilters.UniqueMappedReadsFilter |  |
| ►C**Serializable** |  |
| Cjaligner.matrix.Matrix |  |
| Cjaligner.Sequence |  |
| ►C**WindowProcessor** |  |
| Cumms.core.model.score.WindowProcessor< S extends WindowScore >.AbstractProcessor< S extends WindowScore > |  |


---

Generated on Fri Aug 15 2014 15:11:16 for ESAT by  

 1.8.7
